# Laser-Fabricated Reduced Graphene Oxide Memristors

**DOI:** 10.3390/nano9060897

**Published:** 2019-06-19

**Authors:** Francisco J. Romero, Alejandro Toral-Lopez, Akiko Ohata, Diego P. Morales, Francisco G. Ruiz, Andres Godoy, Noel Rodriguez

**Affiliations:** 1 Pervasive Electronics Advanced Research Laboratory, University of Granada, 18071 Granada, Spain; atoral@ugr.es (A.T.-L.); franruiz@ugr.es (F.G.R.); agodoy@ugr.es (A.G.); noel@ugr.es (N.R.); 2 Department of Electronics and Computer Technology & Center of Research in Telecommunications and Information Technologies, University of Granada, 18071 Granada, Spain; diegopm@ugr.es; 3 Institute of Space and Astronautical Science, Japan Aerospace Exploration Agency, Kanagawa 252-5210, Japan; ohhata.akiko@jaxa.jp; 4 Biochemistry and Electronics as Sensing Technologies Group, University of Granada, 18071 Granada, Spain

**Keywords:** memristor, graphene oxide, laser-scribing, neuromorphic, flexible electronics

## Abstract

Finding an inexpensive and scalable method for the mass production of memristors will be one of the key aspects for their implementation in end-user computing applications. Herein, we report pioneering research on the fabrication of laser-lithographed graphene oxide memristors. The devices have been surface-fabricated through a graphene oxide coating on a polyethylene terephthalate substrate followed by a localized laser-assisted photo-thermal partial reduction. When the laser fluence is appropriately tuned during the fabrication process, the devices present a characteristic pinched closed-loop in the current-voltage relation revealing the unique fingerprint of the memristive hysteresis. Combined structural and electrical experiments have been conducted to characterize the raw material and the devices that aim to establish a path for optimization. Electrical measurements have demonstrated a clear distinction between the resistive states, as well as stable memory performance, indicating the potential of laser-fabricated graphene oxide memristors in resistive switching applications.

## 1. Introduction

The memristor (memory-resistor) is the fourth element needed to complete the relationships between the four fundamental electrical magnitudes (voltage, *v*; current, *i*; charge, *q*, and flux, *ϕ*), which establishes the missing link between charge and flux [1]. The memristor was predicted almost 50 years ago, fundamentally as a theoretical device [2], and then remained forgotten until the last decade when it was physically implemented [3]. Since then, the interest and research devoted to this electrical element have experienced an exponential rise [4,5,6,7]. On one side, it has become one of the most promising candidates for the so-called storage class memories in the form of resistive random access memories (ReRAMs) [8,9,10,11]. On the other side, memristors are devices able to mimic the behavior of biological synapses, opening the path for the future development of artificial intelligence in the emerging field of neuromorphic electronics [12,13,14]. The spike-timing-dependent plasticity (STDP) is the key characteristic in the development of neuromorphic computing circuits [4]; the conductance can be changed by the input pulses (related to the input charge injected into the device), in such a way, that they behave like solid-state artificial electronic synapses [15]. Moreover, memristor-based synapses are simpler and energetically more efficient than those based in complementary metal-oxide-semiconductor (CMOS) emulation circuits, allowing them to pave the way for the next generation of computing and human-machine interfaces.

In contrast to the other existing electrical elements, memristors present a unique voltage-current relationship determined by a closed hysteresis loop pinched in the origin. This characteristic can be considered as the electrical signature of memristors to the point that any device presenting this electrical behavior can be assimilated as one of them. Memristors present an intrinsic non-volatile memory effect because their resistance depends on the history of the device according to a certain state equation [2]: their state of resistance (typically high resistance state, HRS, or low resistance state, LRS) remains unaltered even in the absence of any energy supply. This memory effect has already reached the market and is being used in commercial digital applications [16].

One of the crucial issues that science needs to address concerning the rise of memristive electronics, especially neuromorphic applications, is the selection of the best technology for the fabrication of the memristors. Memristors are commonly classified into four categories depending on the physico-chemical mechanism responsible for the memristive hysteresis: nanoionics, phase-change, electronics (ferroelectrics and those based on charge trapping/detrapping), and nanomechanics [4]. Experimentally, any device from these first three categories applies to neuromorphic electronics due to its “analog” memristive behavior. It has been demonstrated that a large number of solids present analog memristive switching characteristics, including solid electrolytes (such as GeSe or Ag_2_S), perovskites (e.g., SrZrO_3_), transitions oxides (such as NiO or TiO_2_), organic materials, or even amorphous silicon [4]. However, despite the large spectrum of existing memristive materials, there is not yet a real competitor for traditional Silicon-CMOS technologies that support the digital von-Neumann computational archetype. Research on memristive circuits needs to investigate differential advantages over silicon to gather pace within the field of electronics. This means that, apart from demonstrating their particular functionalities, memristors should be based on a cost-effective fabrication approach that avoids the use of scarce materials.

With the rise of graphene and related nanomaterials [17], a new path for the development of inexpensive resistive-switching devices has been opened. Graphene derivatives are being considered, both, for the application at the electrodes of the memristors [18], and as part of active memristive materials [19]. The reader can find a comprehensive review of the state of the art of graphene and transition metal dichalcogenides (TMDs) based resistive switching devices in reference [20]. This new family of memristors can benefit from the intrinsic characteristics of graphene-related materials (i.e., abundant raw materials, structural flexibility, and transparency). Among these emerging candidates, it is graphene oxide (GO), a non-stoichiometric polycrystalline form of nanographene decorated with oxygen-containing functional groups [21], where most of the research interest is focused due to its easy processability and low cost. GO-incorporating memristors can be fabricated with a wide range of techniques, from the deposition of the pristine material [22] to reduced versions of it synthesized by chemical, electrochemical, thermal annealing, microwave irradiation, or photothermal treatments [23]. Among all the aforementioned techniques, the use of high-precision lasers to reduce GO is one of the most attractive alternatives since it allows for the combination of lithography and conductivity modulation in one single environmentally friendly step [24].

The application of laser-reduced graphene oxide for the fabrication of resistive switching devices was introduced first in 2014 by Tian et al. [25]. In that work, the authors presented a low-cost approach targeting the production of random-access memory using laser-reduced graphene oxide as the bottom electrode of a GO-HfO_x_-Ag memristive stack. However, in [25] the authors concentrated on achieving a high-conductivity GO-based electrode while the active memristive material remained as the HfO_x_ itself. In this work, for the first time, we introduce the development of laser-fabricated GO memristors that fully rely on the laser-reduction process to create the active switching element. This new process for the fabrication of memristors takes an inexpensive graphene oxide colloid as the precursor material. A computer numerical control (CNC) driven laser is used to photothermally reduce the GO, altering its nanostructure and leading, under appropriate laser-scribing conditions, to memristive behavior. This fabrication approach is extremely simple, which allows for an intrinsic lithography of the devices. The results have shown that the devices present a promising and stable memristance which is correlated with the formation of low-resistance conductive paths originated by oxidation-reduction reactions within the GO body. The paper is structured as follows: the materials, experimental setup, and fabrication process are described in Section 2. Section 3 presents results on the structural and electrical characterization of the laser-fabricated memristors, showing that these devices point to potential applications not only in non-volatile memory storage but also in analog computing since they present, both, clear discrimination between the resistive states and stable memory performance. Section 4 includes a discussion of the origin of the memristance of the reduced-GO elements, and, finally, the main conclusions are highlighted in Section 5.

## 2. Materials and Methods

### 2.1. Materials

The precursor material for the fabrication of the memristors was an in-house prepared graphene oxide colloid (4 mg/mL). We followed a modified version of Hummers and Offerman’s method [26] starting with the oxidation of graphite for about two hours in an ice bath, using concentrated sulfuric acid (H_2_SO_4_), sodium nitrate (NaNO_3_), and potassium permanganate (KMnO_4_) as functionalization and oxidizing reagents (from Sigma-Aldrich Corp., St. Louis, MO, USA). The oxidized graphite is filtered (HCl) and washed (H_2_O) to remove remaining ions. Then, with the aid of sonication (30 min) and due to the increased interlayer distance caused by the introduction of the functional groups, water molecules can easily penetrate in the layers producing layer splitting leading to the GO colloid. Further details on the production of the GO colloid can be found in [24]. Polyethylene terephthalate films (PET) (3M, St. Paul, MN, USA) were used as supporting substrates for the samples. Electrical access to the memristors was achieved by applying micro-drops of conductive carbon-based paste (Bare Conductive Electric Paint, London, UK), or Ag-loaded conductive paint (RS, Corby, UK).

### 2.2. Experimental Setup

**Figure 1:**
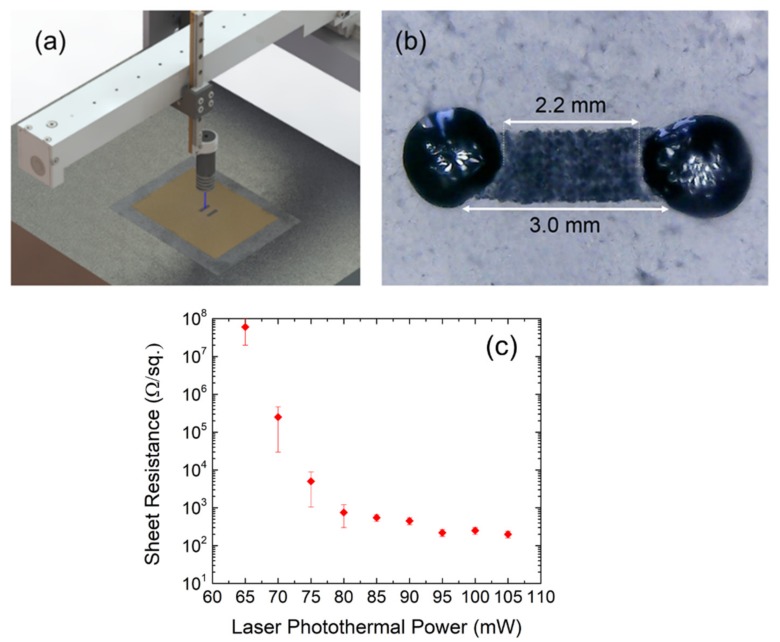
(**a**) Schematic of the CNC-driven laser used for the fabrication of rGO memristors. The spatial resolution of the system used in this work is 10 µm. (**b**) Actual picture of one of the laser-fabricated memristors (L = 2.2 mm, W = 1 mm) using microdrops of bare conductive electric paint^TM^ as contacting electrodes. (**c**) Sheet resistance of laser-reduced graphene oxide samples (4 mg/mL) on PET treated at different laser powers (λ = 405 nm) extracted by the transmission line method (TLM) [27]. Error bars were calculated as the standard deviation of 15 different samples for each laser power.

The CNC driving the laser used for laser-scribing the devices was partially developed in our group. This system allows the patterning of surfaces located in a horizontal holder at 6 cm from the exposure source. The laser excursion speed was fixed at 3 min/cm^2^. The 405 nm laser head was acquired from Q-BAIHE^TM^, model 405ML-300-2290 (Shenzhen, China) using a laser diode as an exposure source (Figure 1a). The power of the laser can be modulated from 10 mW to 300 mW. The 3D-shaker (Seoulin Bioscience, South Korea) used for the GO homogenization during the water evaporation process was set to an orbital speed of 0.3 r.p.s. Scanning electron microscopy (SEM) images were acquired by a field-emission scanning electron microscope (NVision40 from Carl Zeiss, Oberkochen, Germany) set at an extraction and acceleration voltage of 5 kV. Raman spectra were recorded with a dispersive micro-Raman spectrometer (JASCO NRS-5100, Easton, PA, USA) with a green diode as the excitation source (Elforlight G4-30; Nd:YAG, λ = 532 nm). The X-ray photoelectron spectroscopy (XPS) experiments were carried out on a Kratos Axis Ultra-DLD (Manchester, UK), using an X-ray (Al Kα, hν = 1486.6 eV) power of 450 W in a vacuum chamber where the pressure was kept below 10^−10^ Torr. The Casa-XPS^®^ software (Teignmouth, UK) was used for the XPS spectra deconvolution. Attenuated total reflectance Fourier transform infrared (ATR-FTIR) spectroscopy was carried out using a Bruker Tensor 27 spectrometer (MA, USA). The electrical measurements were performed by a Keysight^®^ B2902A precision source-measure unit controlled by Easy-Expert^®^ software (CA, USA). Samples were located on an Everbeing^®^ C-series analytical probe station (Taiwan). 

### 2.3. Laser-Reduced Memristor Fabrication

The raw film for the fabrication of the memristors was prepared by drop-casting the GO colloid on PET films (0.5 mL/cm^2^). Next, they were placed on a 3D-shaker for 48 h at room temperature (relative humidity 50%) until the water was fully evaporated. According to the Kelvin measurements, the resulting GO layer was essentially an electrical insulator (ρ_s_ > 10 MΩ/sq) with an approximate thickness of 200 µm according to the optical profilometry results.

The memristors were fabricated by laser-scribing the surface of the GO film (Figure 1a). The laser is only used for the fabrication of the samples and it is not involved in the electrical measurements carried out for the characterization of the resistive switching of the devices. Starting with the bare GO film, the laser triggers a photothermal reduction process that removes oxygen-containing functional groups (increasing the atomic percentage of carbon) and partially recovers the sp^2^ hybridized carbon-carbon bonds, increasing the conductivity of the nanographene flakes according to the laser power [28]. The laser power can be adjusted to modulate the conductivity of the samples leading to different levels of reduction. The dependence of the sheet resistance of the laser-reduced GO as a function of the photothermal power was investigated. The results, summarized in Figure 1c, show that an increase of the laser power results in a dramatic decrease of sheet resistance (from >MΩ/sq. to <kΩ/sq.) for a relatively narrow power window, reflecting the progressive reduction of the GO sample. It is also possible to observe that, for a laser power below 85 mW, the variability of the sheet resistance increases partly due to the appearance of the resistive switching. For a laser power over 85 mW, the improvement of the conductance (decrease of the sheet-resistance) tends to saturate. 

For the purpose of the experiments, memristors with a macroscopic rectangular size of L = 3 mm, W = 1 mm, were fabricated at a laser power ranging from 40 mW to 100 mW. The laser-scribing process was performed at room temperature with a controlled relative humidity of 50% and a constant airflow forced by a fume extractor system. These conditions avoid any intentional doping during the lithographic process; however, the intentional doping/functionalization of the devices by setting an appropriate environment during the laser-scribing process [28,29] constitutes a path to be explored for boosting the performance of this type of devices. Micro drops of bare conductive electric paint^TM^ or Ag-based conductive paint were placed as electrodes to define the electrical access and to avoid any damage on the active GO material when contacting the devices with probes. This contact approach led to a reduction of the effective length of the devices of about 0.8 mm, resulting in an effective dimension of L = 2.2 mm, W = 1.0 mm (Figure 1b). As observed, the edges of the final device present a slight roughness consequence of the mechanical limitations of the experimental setup. However, this fact should not represent any constraint for scaling down the device if a more sophisticated positioning system is used (i.e., a galvanometric positioning allows a linewidth of 10 µm and a repeatability of 1 µm). 

## 3. Results

### 3.1. Structural Characterization

**Figure 2:**
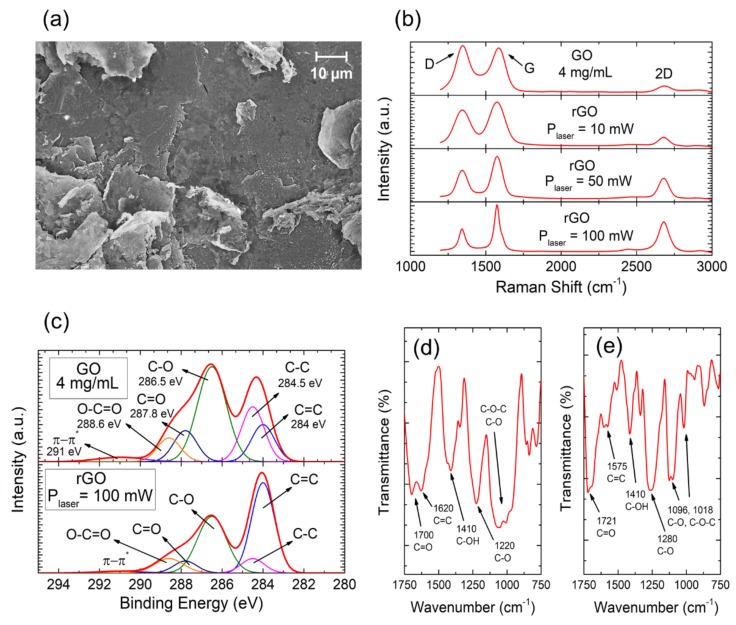
Structural characterization. (**a**) SEM image of laser-reduced graphene oxide at 70 mW (initial GO colloid concentration 0.5 mL/cm^2^). (**b**) Raman spectra acquired from the GO film before and after the laser-assisted reduction for different laser powers. (**c**) Comparison of the C1s peak from the XPS spectrum of both GO (top) and laser-reduced GO (bottom) samples. (**d**) ATR-FTIR spectra of unreduced GO. (**e**) ATR-FTIR spectra of 100 mW laser-reduced GO.

We investigated the changes induced by the laser photothermal process in the chemical structure of the GO (Figure 2) by SEM, Raman spectroscopy, XPS, and ATR-FTIR spectroscopy. 

Figure 2a shows an SEM image of a laser-reduced GO area. The laser-treated surface is characterized by a large roughness and porosity as a consequence of the spontaneous and violent release of oxygen-containing functional groups during the photothermal process.

The Raman spectroscopy provided information about the lattice structure of the sp^2^ hybridized carbon systems. It is worth mentioning that the spectra were acquired with special caution to avoid any undesired photothermal reduction of the GO by the laser stimulation source itself [30]. In particular, the laser power was set to 5.3 mW with an 80% attenuation and acquisition time of 15 s. As can be seen in Figure 2b, the Raman spectra of the bare GO and laser-reduced GO samples are composed of three main peaks at approximately 1,350 cm^−1^ (D), 1,620 cm^−1^ (G) and 2,700 cm^−1^ (2D). The presence of disorders in the sp^2^ structure is evaluated through the relative intensity of the I_G_/I_D_ ratio, while the intensity of the 2D band gives information about the number of layers of the graphene-derived structure (which may range from the one single layer of pristine graphene to the multilayer structure of graphite) [31,32]. Thus, Figure 2b shows the evolution of defects in the graphene-based samples as well as the changes in their layer structure as the photothermal power of the laser increases. For the raw GO, the prominent intensity and area of the D peak, with respect to the G peak, is a consequence of the structural imperfections created by the oxidation process of the graphite. Besides, the almost nonexistent 2D band indicates the multilayer and defective nature of the GO sample. When the laser-scribing process is applied on the surface, the photothermal reduction takes place, and as the laser power increases, the G and D peaks become narrower and the I_G_/I_D_ ratio increases, demonstrating the decrease of the number of defects, and therefore the reduction of the GO. Moreover, the gradual restoration of the crystallographic structure is also manifested by the decrease of the I_G_/I_2D_ ratio along the photothermal process [24].

XPS experiments were performed for a better understanding of the nature of the defects. The results showed that the initial atomic percentages of ~52% and ~45% for both carbon and oxygen species of GO became ~72% (C) and ~26% (O) after the reduction at a laser power of 100 mW. Furthermore, Figure 2c shows the C1s XPS high-resolution spectra of the GO film before and after the laser treatment and their deconvolution. The defects in the structure of the sp^2^ hybridized carbon bonds (284 eV) are associated to C–C sp^3^ bonds (284.5 eV), carbon-oxygen compounds (C–O, 286.5 eV) present as hydroxyl (C–OH) and epoxy (C–O–C) groups, carbonyl groups (C=O, 287.8 eV) as well as O–C=O (288.6 eV) and π-π* transitions (291 eV) [33,34]. Therefore, the GO film presents a large contribution of sp^3^ hybridized carbon bonds and different carbon-oxygen compounds consequence of the oxidation process. The laser treatment turns a large portion of sp^3^ carbon into sp^2^ hybridized carbon, which is a clear indication of a graphene-based composite material [35] and removes a significant portion of the defects introduced by carbon-oxygen compounds, as it was reflected in the Raman results.

All these results are also consistent with those achieved from the ATR-FTIR experiments, shown in Figure 2d,e. In the GO spectrum (Figure 2d), the different oxygen-containing functional groups can be observed at the broad peaks located at 1,700 cm^−1^ (C=O stretching vibrations in carbonyl and carboxyl groups), 1,410 cm^−1^ (C–OH hydroxyl), as well as at 1,220 cm^−1^ and at the band from 968 cm^−1^ to 1,060 cm^−1^ associated with epoxy and other C–O groups. Moreover, one band at 1,620 cm^−1^, due to the skeletal vibrations of unoxidized sp^2^ hybridized carbon bonds, is also observed [36,37,38]. After the laser photothermal reduction (Figure 2e), the sp^2^ hybridized carbon domain is also present but shifted to 1,575 cm^−1^ [39]. Besides, a significant reduction of the bands associated with the oxygen-containing groups is detected, which is clear evidence of a successful laser-reduction of the GO.

### 3.2. Electrical Characterization

**Figure 3:**
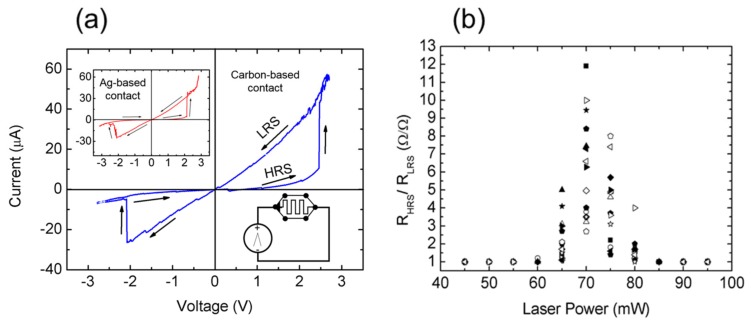
Electrical performance of a laser-reduced GO memristor (P_laser_ = 70 mW, L = 2.2 mm, W = 1 mm). (**a**) Current-voltage characteristic of a memristor showing the characteristic signature of memristance. The voltage has been scanned from −3 V to 3 V with a voltage step of 10 mV. The scanning rate was adjusted to 2 V/s. The top inset of the figure shows an example of another device fabricated with Ag-based contacts. (**b**) Ratio of the resistance measured in the high resistance state (HRS) and low resistance state (LRS) of a set of laser-lithographed graphene oxide memristors fabricated at different laser power (15 devices for each laser power). The resistance was extracted in the range [−1,1] V of the current-voltage characteristics. The memristors were of identical dimensions with an effective length of 2.2 mm and width of 1 mm.

The laser-reduced graphene oxide memristors were initially characterized by cyclically applying a triangular voltage bias at the two surface electrodes [−3.5, 3.5] V and simultaneously monitoring the driven current by a source-measure unit (SMU), as the inset of Figure 3a shows. Unless otherwise specified, the experiments presented hereinafter were performed using bare conductive electric paint^TM^ as the contacting electrode. Representative results of the current-voltage characteristics are shown in Figure 3a for a memristor lithographed at a laser power of 70 mW. As can be observed, the device presents the unequivocal fingerprint of a bipolar memristor [40], corresponding to a closed pinched hysteresis loop collapsed in the origin in its current-voltage characteristic. In Figure 3a, the two resistive states of the device are identified presenting a resistance, in the initial cycle, of R = 896 kΩ and R = 75 kΩ at the HRS and LRS, respectively. These resistances have been measured within the voltage range [−1, 1] V. All the fabricated devices presented a forming-free resistive switching [23]. The top inset of Figure 3a shows an example of another device contacted with Ag-loaded conductive paint. As observed, the device presents similar memristive hysteresis. This contact independence of the memristance allows one to elucidate the underlying mechanisms responsible for the resistive switching as discussed in detail in Section 4 and opens this technology to the investigation of a large variety of contact approaches.

As it is the case of some other reported symmetric memristors [41,42,43], the polarity of the device can only be determined after the first cycle of its current-voltage characteristic. Prospective studies are to be conducted to disclose if it is possible to induce a specific polarity during the fabrication process or by using an electrical approach. The memristive behavior of the devices (i.e., pinched hysteresis loop, as shown in Figure 3a) was investigated for different values of the laser power. The results, summarized as the ratio between the resistive states, are shown in Figure 3b. As observed, the largest hysteresis was spotted for the devices laser-fabricated from 65 mW to 75 mW. Whereas, for the devices reduced at a laser power below 60 mW, the memristance was not observable. This does not mean that resistive switching effects were not taking place in the structures but rather that they were fully masked by the high resistance of the material. At the opposite end of the scale, for devices reduced at a laser power over 80 mW, we did not observe any memristance. We speculate that the number of oxygen-containing functional groups resulting after the laser treatment was too small to appreciate any hysteresis effect on the current-voltage characteristic (see Section 4 for further discussion).

**Figure 4:**
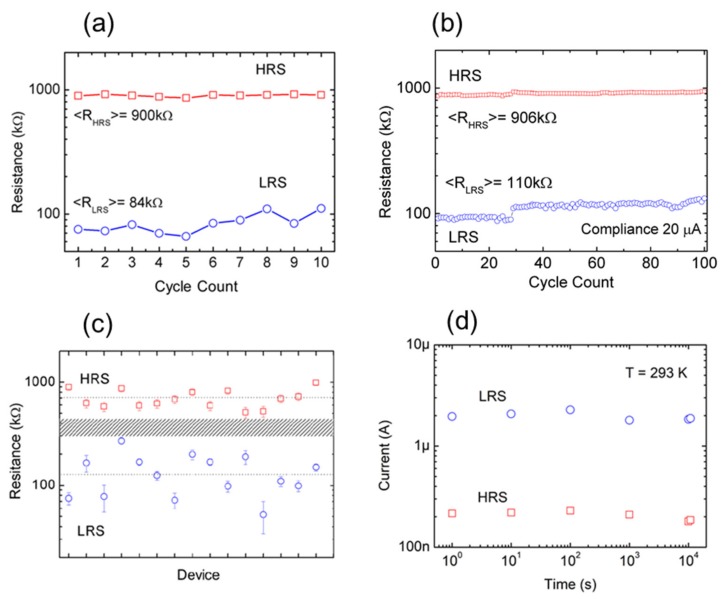
(**a**) Resistance values obtained from successive programming/erasing cycles without current compliance. Average values of the resistance at the HRS and LRS are included. (**b**) Resistance values obtained from successive device cycling demonstrating up to 100 cycles. The experiments were carried out by establishing a current compliance of 20 µA. (**c**) Resistance values obtained from 15 different memristors at the HRS and LRS states. Error bars illustrate the resistance variability of the device during 10 cycles. (**d**) Retention characteristic of laser-reduced graphene oxide memristor at room temperature (same device for LRS and HRS states). The state of the device is read with 0.2 V pulses during 50 ms.

The stability of the resistive states has been assessed by continuous device cycling. Results represented in Figure 4a show that after 10 complete up and down voltage excursions, without establishing any current compliance during the measurements, the states remain reasonably stable with a tolerance <30% and the average values of high and low resistance states <R_HRS_> = 900 kΩ and <R_LRS_> = 84 kΩ, respectively. After the cycling of the device, there is neither a significant degradation in the performance nor an overlap between the HRS and LRS. The devices can safely be cycled further if the current compliance is set to 20 µA, limiting the power dissipated and preventing irreversible resistive switching. As shown in Figure 4b, under these requisites, it is possible to achieve 100 cycles. These values, despite still well below the endurance values of better established inorganic memristor technologies [44] are comparable with other GO-based memristors. For example, in [45] the endurance properties of Al/GO/ITO devices were evaluated during 100 switching cycles, showing that the HRS and LRS values and its ratio maintained stable values. In a recent work [46], the authors make clear statements about the lack of research efforts in organic devices compared with their inorganic counterparts. They specifically highlight that problems arise from insufficient reproducibility, endurance, stability, scalability, and low switching speed. In Table S1 (Supplementary Materials) they present a comprehensive comparison between state of the art organic and inorganic oxide memristors. The data presented for the reduced-GO material indicates an endurance of 250 cycles, which is far below (orders of magnitude) the values already demonstrated for other materials shown in Table S2 of the same work. Specifically, they refer to [47] where a metal/RGO/metal memory device was fabricated and characterized. In that work, after 250 cycles, the HRS and LRS values show a noticeable degradation as depicted in its Figure 8a. In [48] a Pt/GO/ITO device was fabricated and characterized, which demonstrates good retention characteristics with up to 100 cycles.

Figure 4c shows statistical results of the HRS and LRS states of 15 laser-fabricated graphene oxide memristors (lithographed at 70 mW). The variability, especially that of the LRS state, is relatively high; however, there is no overlap in the distributions of states. Furthermore, the resistance ratio between the states remains, on average, around a factor of 6, which can be considered a promising achievement given the early stage of development of the devices (best ratio = 11.9, worst ratio = 3.2). Finally, Figure 4d shows results on the characterization of the retention time of a memristor at room temperature. The current at the HRS and LRS was monitored periodically (every time-decade) applying 0.2 V/50 ms pulses during 4 hours after programming the HRS or LRS exemplifying the non-volatility of the states. A low reading voltage (0.2 V) was selected to avoid device disturbance because in this experiment, it is only necessary to discriminate the states.

## 4. Discussion

An in-depth understanding of the physical mechanisms of the resistive switching-process in GO and reduced-GO is yet a subject under study and not exempt from controversy [49]. Two main models have been proposed to explain the formation of conductive paths involved in the resistive switching of GO. In one of the theories, the conductivity of the GO layer is modified according to redox reactions of the electrodes at the interface between GO and metals [50]. Our results have shown equivalent memristive behavior under different contact approaches (see Figure 3a), making us rule out this process as the origin of the memristance. This is specifically supported because, in the case of the devices contacted with bare conductive electric paint^TM^, there are no metallic agents in the composite (water, natural resin, conductive carbon, humectant agent, diazolidinyl urea, and preservative agents [51]).

The other model to explain the resistive switching in GO films proposes that the conductive path is caused by transforming insulating sp^3^ domains to conducting sp^2^ bonds (oxygen vacancies) based on the detachment of oxygen groups under the action of the electric field [49,52]. Several works based on first principles and statistical calculations [53,54] have demonstrated the tendency of the oxygen functionalities to agglomerate and form highly oxidized domains surrounded by areas of pristine graphene. In [55], the authors studied the reversible oxidation of graphene oxide by DFT. Their studies show that although the bonding between oxygen and carbon atoms is strong, the migration barrier of oxygen atoms is below 0.8 eV. These values are further corroborated in [53], which estimates an energy of 0.6 eV (on average) for those endothermic reactions. 

This second model is likely to explain the switch of the resistance of laser-fabricated graphene oxide memristors. Thus, the memristance relates to a bulk phenomenon involving the drift of oxygen ions and oxygen-containing groups inducing local changes in the level of reduction of the GO turning it into a more conductive state [23,56,57]. This statement is also supported by estimating the energy involved during the resistive switching process. In our devices, the transitions HRS to LRS are typically occurring at a current of 10 µA (Figure 3a) and a resistance (HRS) of 1 MΩ (Figure 4a). Therefore, the power involved in the transition is about ~0.1 mW. The transition time cannot be determined precisely due to the large time constant of the devices which masks the actual physical phenomenon somewhere below 20 ms. However, we do not expect this device to achieve the sub-ms transitions of more scaled technologies [58]. Therefore, we elucidate that this transition is in the range from 1 ms to 10 ms. According to this, the energy supplied to the device is above 10^12^ eV, consistent enough to trigger a significant percentage of the migration and recombination of oxygen ions and functional groups across the basal plane [53], since the number of surface sp^3^ C–O bonds can be roughly estimated in the order of ~10^13^ C atoms (~10^19^ C atoms cm^−2^ in the graphitic structure [59] and 60% of carbon sp^3^ hybridization with oxygen-containing functional groups according to the XPS experiments).

**Figure 5:**
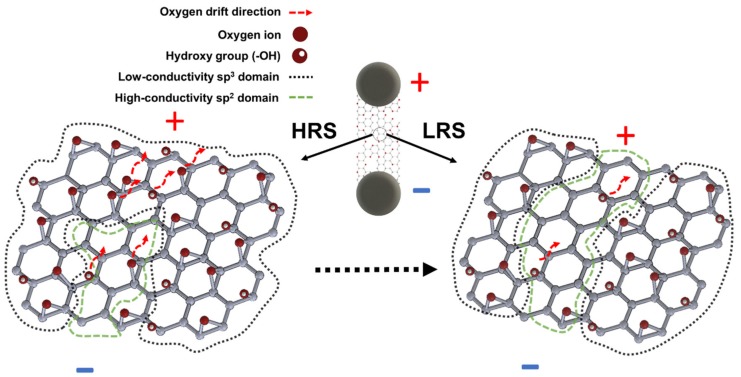
Conceptual model of the origin of the resistive switching of laser-fabricated graphene oxide memristors illustrating the transition from HRS to LRS. Starting from an HRS state, under the action the current flow, the electrostatic potential is mostly applied on the non-conductive sp^3^ domains resulting in large local electric fields. The sp^2^ based conductive domain (area surrounded by the green dashed line) is extended by the field-assisted migration of oxygen ions, creating a local high conductivity path leading to an LRS state.

Figure 5 illustrates the process of resistive switching. The non-uniformity in the number and location of the functional groups of the partially reduced graphene oxide [60,61,62] is responsible for the creation of different conductive domains. sp^2^ high-conductive domains are interrupted by low-conductive ones (sp^3^) at a nanoscale level. When a current is flowing, the electrostatic potential is mostly applied on those non-conductive regions resulting in large local electric fields. sp^2^ based conductive paths are set up under the action of the current flow leading to an LRS state. The hypothesis of the formation of interconnected high-conductivity domains does not mean that the high conductivity state is related to a homogeneously distributed conduction medium, but rather the LRS is correlated with a confined effect associated to the formation of a percolation path (or paths) of highly reduced GO by the energy accumulated in the device. Under reversed polarity, a partial recombination of the oxygen vacancies and mobile ions is possibly resulting in the recovery of the HRS. According to [49], this recombination can also be triggered by thermal effects resulting in degradation of the LRS or even in a retention failure when it is produced spontaneously. This latter statement is in agreement with our need to limit the current driven at the LRS (Figure 4b) when performing multiple cycles to avoid the collapse of the current hysteresis. 

## 5. Conclusions

This work addressed the fabrication of organic memristors based on the laser-assisted reduction of graphene oxide. This technique has been demonstrated as a fast and reliable approach for fabricating the devices with a clear advantage in its simplicity and intrinsic lithography. The results have proved that after tuning the photothermal intensity of the ablation process, the devices feature a prominent and stable memristance. Therefore, this fabrication technique may constitute a new high throughput approach for the advent of memristive circuits. According to our experiments and existing theories, the resistive switching of the devices is attributed to oxygen-containing functional groups drift modifying the local stoichiometry of the reduced-graphene oxide layer. Low-resistance conductive paths are formed in the bulk of the material when sp^3^ domains are tuned into sp^2^ domains (oxygen vacancies). The sp^3^ domains can be recovered by reverse polarity and these mechanisms are likely to be triggered by dissipative effects and assisted by the electric field.
